# Supplementary material for: Human mobility and outbreak origins in epidemic spread: Insights from agent-based modeling
Source: PLoS Comput Biol. 2026 May 12;22(5):e1014279. doi: 10.1371/journal.pcbi.1014279 (PMC13186328; doi:10.1371/journal.pcbi.1014279)
Supplement: S1 File — (PDF) [file pcbi.1014279.s004.pdf]

Descriptions of the algorithms underlying the transCovasim model

Initialization

During the initialization phase, the number of places allocated for tourists is determined for each city as a fraction of the city's population equal to

$$\max\left(0.1, \sum_j \frac{2 \cdot \texttt{timeRelax}}{\texttt{maxMultCoef} \cdot \texttt{adjacencyMatrix}[i,j]}\right),$$

In other words, the number of places allocated to tourists is greater than or equal to twice the mathematical expectation of their number, with the default value of `maxMultCoef` equal to one.

These agents are then added to cities as absent ($\texttt{inCity} = \texttt{False}$) and integrated into social interactions using `touristLayer` in accordance with the specified parameters.

After initialization, the model enters the main simulation cycle.

Function for placing people in the destination city

The function passes all attributes of tourist agents `rest` to agents in the destination city with indices `inds`, except for the identifier `uid`, and then marks their presence in the city ($people.inCity[inds] \leftarrow True$) (Algorithm 1).

Algorithm 1

```
function UpdatePeopleByRest(people, rest, inds)
    for each arrayMember in people.arrayMembers do
        if arrayMember ≠ uid then
            people[arrayMember][inds] ← rest[arrayMember]
        end if
    end for
    people.inCity[inds] ← True
end function
```

Function for placing people who should return to the city

The function returns agents `backPeople` according to their native identifier `backPeople.trueUid` using the function `UpdatePeopleByRest`.

Function for placing tourists who arrived in the city

The function determines the identifiers available for tourist accommodation in the destination city, selects the required number of them corresponding to the number of arriving tourists, if there are enough places, or all remaining places otherwise. Then, agents are placed in the places with the selected identifiers using the `UpdatePeopleByRest` function (Algorithm 2).

Algorithm 2

function AddTourists($people, tourists$)
    $allTouristsInds \leftarrow people.uid[(people.uid \geqslant people.popSize) * (\textbf{not}\ people.inCity)]$
    $freeTouristsInds \leftarrow allTouristsInds[: tourists.popSize]$
    $updatePeopleByRest(people.tourists, freeTouristsInds)$
end function

Function for choosing and extracting tourists who should leave their city

The function corresponding to the daily outflow of tourists from the city `outflowRatioToCities` sets their numbers from a Poisson distribution. It is checked whether there are enough people in the city to send. If there are not enough, the number of tourists to be sent is recalculated according to the number of people in the city. Then, this number of agent identifiers is selected from among the local residents (`people.uid` < `people.popSize`) who are in the city (`people.inCity == True`). This list is divided into lists by destination city based on known transport flow ratios `outflowRatioToCitiesPercent`. For each destination city `cityInd`, severe, critical, and dead agents are removed from the list of tourists. The remaining agents are assigned a duration of stay in another city `people.restInAnotherCityDays`, which is either identically equal to 1 for all when modeling commuting flows, or from a Poisson distribution without 0 otherwise. Then, all departing agents from the `touristPeople` list are extracted from the departure city and stored as a list of pairs (`cityInd`, `touristPeople`) (Algorithm 3).

Function for choosing and extracting people who should return to their city

The function determines the indices of agents whose home city matches the index in the cycle (`people.ownCity == cityInd`), whose travel time has expired (`people.restInAnotherCityDays == 0`), and who are not severe, critical, or dead at the current simulation step. After that, agents with these indices `backPeople` are extracted from the city where they are located and added to the list of returns as a pair (`cityInd`, `backPeople`) (Algorithm 4).

Algorithm 3

```
function ExtractTourists(people)
    peopleLeftCityToCityCounts ← Poisson(popSize ∗ outflowRatioToCities)
    inCityRestrictionInds ← people.uid[people.inCity ∗ (people.uid < people.popSize)]
    if sum(peopleLeftCityToCityCounts) > len(inCityRestrictionInds) then
        peopleLeftCityToCityCounts ← Multinomial(len(inCityRestrictionInds), peopleLeftCityToCityCounts/sum(peopleLeftCityToCityCounts))
    end if
    allPeopleLeftCityInds random selection of sum(peopleLeftCityToCityCounts) indices from 0 to len(inCityRestrictionInds)
    peopleLeftCityToCityInds ← split(allPeopleLeftCityInds, cumsum(peopleLeftCityToCityCounts)[: −1])
    listTouristPeople ← empty list
    for cityInd ← 0 to citiesCount − 1 do
        if cityInd = ownInd then
            continue
        end if
        touristPeopleIndsInCity ← peopleLeftCityToCityInds[cityInd]
        touristPeopleInds ← inCityRestrictionInds[touristPeopleIndsInCity]
        touristPeopleIndsFiltered ← select indices from touristPeopleInds where not (people.severe or people.critical or people.dead)
        touristPeople ← select people with indices touristPeopleIndsFiltered
        if lambda == 0 then
            restInAnotherCityDays ← Ones(touristPeople.popSize)
        else
            restInAnotherCityDays ← ZeroTruncatedPoisson(lambda, touristPeople.popSize)
        end if
        append(cityInd, touristPeople) to listTouristPeople
    end for
    remove people with indices allPeopleLeftCityInds from people
    return listTouristPeople
end function
```

Algorithm 4

```
function ExtractBack(people)
    listBackPeople ← empty list
    for cityInd ← 0 to citiesCount − 1 do
        if cityInd = ownInd then
            continue
        end if
        shouldBackCityInds ← find people who should return to their own city and who is not (people.severe or people.critical or people.dead)
        backPeople ← select people with indices shouldBackCityInds
        remove people with indices shouldBackCityInds from people
        append(cityInd, backPeople) to listBackPeople
    end for
    return listBackPeople
end function
```
