# Supplementary material for: Human mobility and outbreak origins in epidemic spread: Insights from agent-based modeling
Source: PLoS Comput Biol. 2026 May 12;22(5):e1014279. doi: 10.1371/journal.pcbi.1014279 (PMC13186328; doi:10.1371/journal.pcbi.1014279)
Supplement: Attachment — Submitted filename: Response to Reviewers.pdf [file pcbi.1014279.s005.pdf]

Response to the Reviews and Decision

Title: Transportation flows and outbreak origins in epidemic spread: Insights from agent-based modeling

Manuscript Reference Number: PCOMPBIOL-D-25-02107

Authors:
Konstantin A. Klochkov
Ivan E. Kozlov
Elena N. Ilina
Alexander I. Manolov

Date: March 4, 2026

Message from the Authors

Dear Editors and Reviewers,

We appreciate your comments and the opportunity to improve our article. Thanks to your deep dive into the details of the research and the source code of the model and experiments, we were able to make our work clearer to readers unfamiliar with Covasim and the field of agent-based modeling, as well as improve the quality of the experiments.

Below, we respond in detail to each of your comments, indicating the changes we have made to the text of the article. We are also providing a revised version of the manuscript and a version with the changes highlighted. Changes to the code of the experiments are available in our repository on GitHub at `https://github.com/KonstantinKlochkovv/transportation-motifs`.

Thank you for your time and effort in reviewing our article. We hope that our responses and the changes we have made will address your concerns and resolve any issues that have arisen.

Sincerely,
Konstantin A. Klochkov, Ivan E. Kozlov, Elena N. Ilina and Alexander I. Manolov.

Response To Editors

Editors Comment

1) Please ensure that the CRediT author contributions listed for every co-author are completed accurately and in full.

At this stage, the following Authors/Authors require contributions: Konstantin A. Klochkov, Elena N. Ilina, Ivan E. Kozlov, and Alexander I. Manolov. Please ensure that the full contributions of each author are acknowledged in the "Add/Edit/Remove Authors" section of our submission form.

Response

- **Konstantin A. Klochkov:** Conceptualization, Data Curation, Formal Analysis, Investigation, Methodology, Software, Validation, Visualization, Writing — Original Draft Preparation, Writing — Review & Editing;
- **Ivan E. Kozlov:** Data Curation, Methodology, Software, Validation;
- **Elena N. Ilina:** Funding Acquisition, Project Administration, Resources, Supervision;
- **Alexander I. Manolov:** Conceptualization, Formal Analysis, Methodology, Project Administration, Writing — Review & Editing.

Editors Comment

3) Some material included in your submission may be copyrighted. According to PLOSu2019s copyright policy, authors who use figures or other material (e.g., graphics, clipart, maps) from another author or copyright holder must demonstrate or obtain permission to publish this material under the Creative Commons Attribution 4.0 International (CC BY 4.0) License used by PLOS journals. Please closely review the details of PLOSu2019s copyright requirements here: PLOS Licenses and Copyright. If you need to request permissions from a copyright holder, you may use PLOS's Copyright Content Permission form.

Please respond directly to this email and provide any known details concerning your material's license terms and permissions required for reuse, even if you have not yet obtained copyright permissions or are unsure of your material's copyright compatibility. Once you have responded and addressed all other outstanding technical requirements, you may resubmit your manuscript within Editorial Manager.

Potential Copyright Issues:

i) Figures 1A, 2A, 2B, and 3A. Please confirm whether you drew the images / clip-art within the figure panels by hand. If you did not draw the images, please provide (a) a link to the source of the images or icons and their license / terms of use; or (b) written permission from the copyright holder to publish the images or

icons under our CC BY 4.0 license. Alternatively, you may replace the images with open source alternatives. See these open source resources you may use to replace images / clip-art:

- https://commons.wikimedia.org

- https://openclipart.org/.

Response

We have updated the virus clip-art in our figures. All clip-art used in the manuscript (apartment, house, and virus) are now licensed under CC0. For your reference, we provide the corresponding source links:

- **Apartment:** `https://www.svgrepo.com/svg/187275/apartment`;
- **House:** `https://www.svgrepo.com/svg/259343/house`;
- **Virus:** `https://openclipart.org/detail/320041/virus-bacteria-silhouette`.

Editors Comment

4) Please amend your detailed Financial Disclosure statement. This is published with the article. It must therefore be completed in full sentences and contain the exact wording you wish to be published.

1) State the initials, alongside each funding source, of each author to receive each grant. For example: ”This work was supported by the National Institutes of Health (####### to AM; ###### to CJ) and the National Science Foundation (###### to AM).”

2) State what role the funders took in the study. If the funders had no role in your study, please state: ”The funders had no role in study design, data collection and analysis, decision to publish, or preparation of the manuscript.”

3) If any authors received a salary from any of your funders, please state which authors and which funders.. If you did not receive any funding for this study, please simply state: u201cThe authors received no specific funding for this work.u201d

Response

Financial Disclosure statement: This work was supported by a subsidy from Rospotrebnadzor (The Federal Service for Surveillance on Consumer Rights Protection and Human Wellbeing), No. 141-02-2023-208. The funders had no role in study design, data collection and analysis, decision to publish, or preparation of the manuscript.

Response To Reviewer #1

Reviewer Comment

> 1. I think mobility and transportation flow are in different scopes. Please check the difference between these terms. The definition of inbound and outbound flows are not clear as well. It is suggested to rename your title.

Response

We agree with the suggestion. The title of the article has been changed to “Human mobility and outbreak origins in epidemic spread: Insights from agent-based modeling.“ We have also changed the terminology in the text where misunderstandings could arise. However, in most places, the terms “traffic flow“ and “flow“ have been left unchanged because they are commonly used terms in the literature on the spread of epidemics in transportation networks [1–13].

Reviewer Comment

> Introduction:
>
> 2. The existing structure has listed relevant evidence of different epidemics and shown the connection between mobility and disease spreading. However, this part is too redundant. How about different methodologies that help investigate how mobility influence disease spreading? Overall, please state the research gap of developing an agent-based transCovasim model in this study.

Response

We agree that this section required further clarification, so we have revised the Introduction to include a discussion of different modeling approaches for studying the spread of epidemics in transportation networks and to clearly state the research gap that developing our agent-based model transCovasim fills.

> “The spread of infection in transport networks is described by a variety of different models, but conceptually they can be divided into the following groups: compartmental, network-based, and agent-based models. Compartmental models describe the number of people in different states (i.e., compartments, such as susceptible, infected, recovered) using differential equations for each individual settlement. To account for mobility, terms describing the movement of people in different states between cities and the incidence of disease during movement are added to these equations. Such models assume uniform mixing within each population and do not take into account heterogeneity in human behavior. Network-based models represent settlements as network nodes and movements between them as edges, where infection can spread both within nodes and through edges (i.e., population-based models, e.g., reaction-diffusion equation describing the metapopulation system). Individual-based network-based models represent individuals as network nodes, connected in dense social networks within settlements,

and movements between them as edges between these networks. In agent-based models, agents represent individuals that have their own characteristics (e.g., age) and individual disease progression, can infect each other according to a social network, perform various actions and move between different locations [14].

When transitioning from compartmental models to network-based models and from network-based models to agent-based models, we gain the ability to model heterogeneity in human behavior and more complex interactions between people, but this also increases the complexity of the model and the data requirements. In existing agent-based model studies, the task of investigating the impact of human mobility on the spread of epidemics has been addressed using specific, non-general-purpose models or using highly detailed models such as EpiSimdemics [15] and EMOD. This fact points to an existing gap in research and the need for agent-based model with a balanced level of detail and complexity.

We aim to fill this gap with our model transCovasim, an agent-based epidemiological model built on Covasim [16]...“

Introduction

Reviewer Comment

Methods:

3. Please state your transCovasim model by introducing three components 1) agent; 2) environment; 3) interaction rules, which is widely used in the filed of ABM. Please add a technical framework of ABM.

4. the rule of transmission dynamics of deases should be better explained in the transCovasim model.

Response

We agree that the manuscript lacked a description of the underlying model, Covasim. Its description has been added as a separate subsection in Methods. Also, before the detailed description in Methods and Supplementary File 1, a brief description of transCovasim and the modifications required to adapt the basic model for studying human mobility in an agent-based framework were added.

“Covasim [16] is an open-source agent-based model that incorporates realistic population structure, disease progression, and interventions. In Covasim, each agent (one person or a group of people, depending on the population scale) can be in one of these states: susceptible, exposed, infectious (divided into asymptomatic, presymptomatic, mild, severe, critical), recovered, and dead. The duration of each phase of the disease is determined individually for each agent from a lognormal distribution with parameters taken from COVID-19 studies. The probabilities of developing symptoms, severe symptoms, developing into a critical case, or dying, relative susceptibility vary for agents depending on their age, and these data are also taken from COVID-19 studies.

Agents are grouped into a social network containing households, schools, workplaces, and community contacts built using age-specific demographic data.

Transmission of infection can occur when there is contact between an infected and susceptible agent. The probability of transmission depends on the type of contact (household, workplace, school, community contact) and can be modified by interventions such as social distancing. In addition to varying susceptibility to infection depending on the age of the agent and the probability of transmission through contact, the infectiousness of agents is also individual and is determined by the distribution of viral load. The time dependence of the viral load in Covasim consists of two stages: an initial short stage with a constant high viral load, followed by a longer stage with a constant lower viral load. The viral load adjusts the probability of transmission through contact on a daily basis throughout the entire period of the agent's infectivity.

Each Covasim run consists of several stages. First, a simulation object is created and all necessary parameters are loaded. Then, agents are created according to age distribution data and connected into a contact network according to the selected method of synthetic population generation. Then, in a cycle at each time step, the population is scaled, i.e. dynamic rescaling, (if necessary to improve performance); health system constraints are applied; agent states are updated, including those related to the development of infection; random agents are infected (importation events); interventions are applied; the probabilities of further infection through the contact network are calculated; and the resulting metrics are calculated."

Methods, Covasim

"transCovasim is a framework for studying human mobility in agent-based models of epidemic spread. It organizes the transfer of agents between parallel simulations of the base agent-based model while preserving the initial social network structure within each population. The interaction between tourists and city residents is implemented using a separate social layer, and four additional agent attributes are added to ensure the movement of agents. transCovasim follows the same rules of infection transmission dynamics as the base model. In this work, the Covasim model [16] was taken as the base agent-based model."

Methods, transCovasim

Reviewer Comment

> Have you considered the heterougeneity brought by mobility through different transportation modes? If no, why you select ABM as your method.

Response

We are grateful that the Reviewer raised this question. We did not consider the presence of several modes of transport simultaneously in any of our scenarios. However, the properties of the transport flows under consideration were different: in experiments with two cities, the duration of tourist trips was determined from a zero-truncated Poisson distribution with an average of 7 days, and the transmissibility multiplier through their contacts corresponded to the community contacts layer in the Covasim [16], while in the experiments with a hub and satellites, the trip duration was fixed at 1 day, and the transmissibility multiplier corresponded to the workplace contacts layer in

the Covasim [16]. Also, in our model, agents cannot leave their city or return if they are in a severe, critical or dead state, which we believe is necessary to consider when modeling epidemics. It is for these reasons that we chose ABM as our modeling method.

Reviewer Comment

> Design of the experiments:
>
> 5. This study has three scenarios: 1) two-city transport model with equal/unequal agent counts; 2) hub-and-satellite transport model and 3) detecting the outbreak's origin in the hub-satellite model. Please state the reason why you designed these experiments.

Response

We agree that the rationale for selecting the considered scenarios required further elaboration. We have added an explanation of the rationale for designing these experiments in the Methods section.

> "Our work includes a study of four scenarios of epidemiological computational experiments. We constructed these scenarios by gradually increasing the complexity of the model and approaching the main task of detecting the outbreak's origin. The first scenario (two-city transport model with equal agent counts) is a minimal model for determining the rate of infection spread between cities in the absence of asymmetry in flows, with the aim of examining the influence of flow magnitude and infection transmissibility. The second scenario (two-city transport model with unequal populations) complicates the model by introducing asymmetry in transport flows to analyze the impact of flows in different directions on key epidemiological metrics (cumulative and peak infections, peak day of infections). The third scenario (hub-and-satellite transport model) introduces a more realistic transport model containing a large central city and four smaller satellite cities connected to the central city by large commuting flows. This scenario allows us to investigate how the city where the epidemic began (hub or satellite) affects the course of the epidemic and the effectiveness of transport flow restrictions. The fourth scenario (detecting the outbreak's origin in the hub-satellite model) focuses on the main task of our work, which is to identify the outbreak city based on time series of the number of infections in cities in the hub-satellite model with different transport flow multipliers and population testing probabilities."
>
> **Methods, Design of the experiments**

Reviewer Comment

> Results:
>
> 6. how about the validation of your model?

Response

Following the Reviewer's recommendation, we have added a separate subsection in Methods describing the validation of the Covasim and transCovasim models.

> "Covasim disease progressing parameters such as relative susceptibility to infection, probabilities of developing symptoms, severe symptoms, critical case, infection fatality ratio as well as parameters for default viral load distribution were validated from model fits to data on numbers of cases, numbers of people hospitalized and in intensive care, and numbers of deaths from Washington and Oregon states, USA and international estimates [16]. Moreover, Kerr et al. [16] showed that the Covasim model is able to reproduce the three distinct infection waves and temporal changes in test positivity rates during calibration period in King County, Washington, USA. During the prediction period, the model demonstrated correct prediction of the trend in cases and underestimated the number of deaths, however the data was still in the 80% forecast interval.
>
> In the transCovasim model, we additionally validated the algorithms underlying human mobility: we checked the constancy of the total number of agents in simulations, the restriction on movement for agents in severe, critical, and dead states, the consistency of actual flows between cities with those specified in the parameters, and the return of tourists to their city of origin after the end of their trip. In addition, for each simulation, we added 15 simulation steps before seeding the infection to achieve a steady state in simulations where multidirectional transport flows are not balanced, and demonstrated the sufficiency of this period (S3 Fig)."
>
> **Methods, Model validation**

Reviewer Comment

7. how did you design restriction strategies?

Response

We agree that the design of the restriction strategies required further explanation. We have added a paragraph in the Methods section explaining the choice of restriction strategies and their parameters.

> "Restriction strategies for hub-satellite experiments were designed as follows: from 31 January 2020 to March 2020 actual mobility from Wuhan to other Chinese cities was 15 to 20 times smaller than expected volumes [4], number of international tourists decreased by 58-78% due to COVID-19 [8]. We therefore considered 10-fold restriction in flows as a realistic strict scenario, and 100-fold restriction as an extreme scenario. Reducing flows only on links incident to the outbreak city is a more targeted strategy, while reducing all flows is a more general strategy that does not require identifying the outbreak city. We introduced restrictions at various times to evaluate the importance of timing for intervention effectiveness."
>
> **Methods, Design of the experiments**

Response To Reviewer #2

Reviewer Comment

I have a few technical questions for author consideration:

→ Dynamic rescaling. Covasim has a ‘rescale‘ parameter that is set to True by default. This parameter configures dynamic rescaling. In Covasim, there is only one population, and all agents have the same scaling factor. With dynamic rescaling, the effective scale of agents increases over time. Conceptually, this process represents ”zooming out” from an initial small seed area to the full population being simulated.

The implementation here is quite clever. While Covasim runs a single process, the authors have extended the MultiSim capabilities using multiprocessing ”barrier” and ”lock” to synchronize. However, with this modification, there are multiple instances of the simulation running in lockstep, but each instance may have a different population scaling factor. An agent migrating from one city to the next may have a different scale factor.

The scale factor increases following seeding, but quickly stabilizes at a maximum value – so it might be a non-issue. Please check, and consider rerunning the simulation experiments with rescaling disabled.

Response

Thank you very much for this comment. Dynamic rescaling is indeed enabled by default for individual Covasim simulations and was not disabled by us in the experiments in our manuscript. Despite the rapid stabilization of the scale factor, the tourist scales could have been different for different cities for different simulation steps. In light of this, we reran all experiments without dynamic rescaling and updated the results in the paper.

Reviewer Comment

→ I wonder about population sizes. Each node starts with a specified population size, but the number of agents in each city will change dynamically as migration unfolds (unless balanced?).

• If not balanced, consider adding a supplementary figure showing how the population in each city changes throughout the course of a simulation.

• Worth checking that the upper limit of population is not reached.

• Additionally, it might be worth letting between-city mobility (population sizes) reach steady state before seeding infection.

- Finally, ensure that key statistics like incidence and prevalence are calculated correctly with dynamic city sizes.

Response

As the Reviewer correctly points out, it is reasonable to allow population sizes to stabilize before seeding infection, so when rerunning the experiments, we added a period of 15 simulation steps without infection in the population. We added Supplementary Figure 3, which demonstrates the change in the number of people in cities over time and shows that 15 days is a sufficient stabilization period for our experiments. It is important to note that the curves in this figure show the deviation in the number of people in the city from its median across simulation days for a given random seed. In the scenario with two identical cities (panel A), the flows in different directions are equal, so the number of people in the city remains constant during the stabilization period. Deviations from 0 are due to the fact that later during the epidemic, people in severe, critical, and dead states are prohibited from moving between cities, and the course of the epidemic itself is stochastic. In the scenario with two different cities (panel B), the flows are unbalanced and stabilize in about 7 days, which is equal to the average time an agent spends in the destination city. In the hub-satellite scenario (panel C), the duration of travel between cities is 1 day, so no stabilization period is required. When restrictions on human mobility are introduced, some people remain in the satellite instead of traveling to the hub, so the median number of people in the satellite increases and in the hub decreases. This is the reason for the observed shift of the curves towards lower values for satellites and higher values for the hub. In the detection scenario in the hub-satellite system (panel D) the duration of travel between cities is 1 day, and transport flows are constant throughout the simulations, so no stabilization period is required.

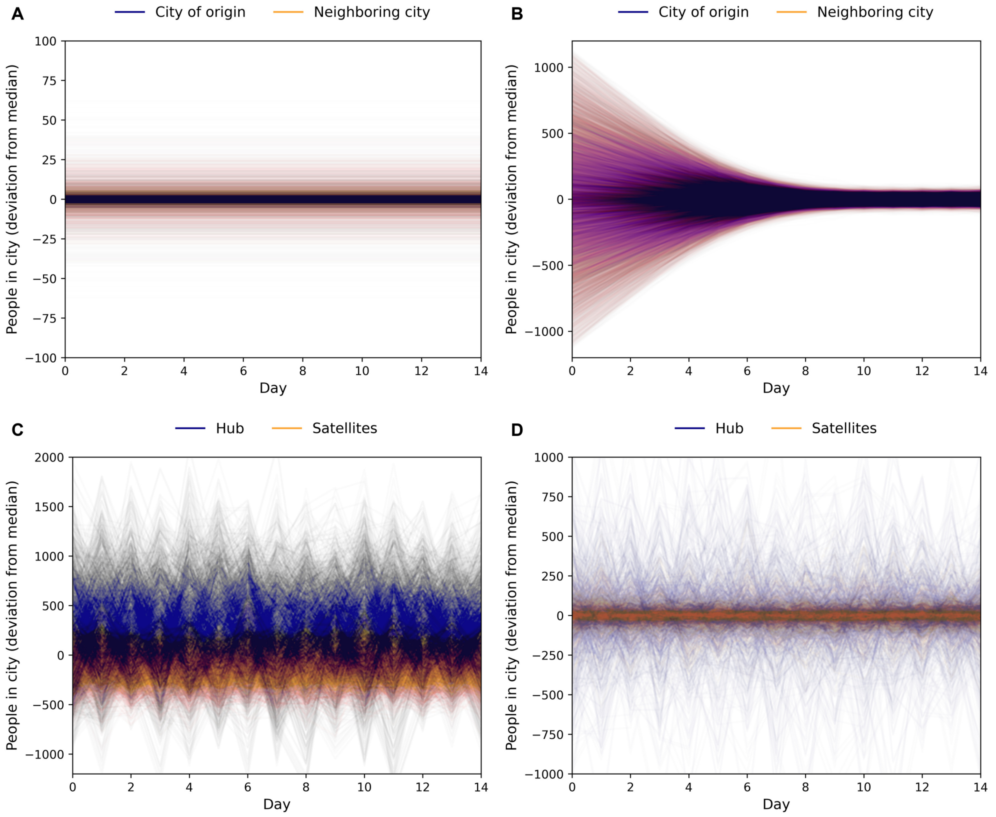

S3 Fig. Stabilization of the number of agents in cities. A–D: Time series of the deviation of the number of agents in each city during the stabilization period 15 steps before seeding infection from the median value for all time steps of a specific simulation for two-city system with identical populations (A), two-city system with unequal populations (B), hub-satellite system (C), and hub-satellite system with outbreak origin detection (D).

In this work, we did not consider effects associated with overcrowding of tourist sites within cities. In the current implementation of transCovasim, such scenarios lie outside the supported operating range of the model. Under the default setting, where the number of tourist sites is set to twice the expected number of tourists in the city, this limitation is not encountered.

Incidence and prevalence were previously calculated relative to the initial population size of cities. We recalculated these metrics, taking into account the current population size of the city with tourists at each simulation step.

Reviewer Comment

> → The mixing structure within each city is in fact quite detailed in these simulations. I did not realize this until looking at the source code. The populations are built using the SynthPops package configured for Seattle (for which rich microdata are available). It might be worth a sentence or two in the methods section describing that agents have static home/school/work/community connections that are not affected by between-city migration.
>
> → The approach to moving agents between cities, each running in a separate simulation, is already quite novel! I might suggest that the authors describe that agents are physically moved, not the a rewiring of the network. This pattern could be reused by others, so highlighting code availability and key changes from Covasim would be welcome.

Response

Thank you for pointing this out. We have updated the Methods and Data and Code Availability Statement sections and highlighted the special features of the transCovasim software implementation. In addition to a detailed description of the modifications required to adapt individual Covasim simulations to our framework, a brief description of them has been added to Methods.

> "transCovasim is a framework for studying human mobility in agent-based models of epidemic spread. It organizes the transfer of agents between parallel simulations of the base agent-based model while preserving the initial social network structure within each population. The interaction between tourists and city residents is implemented using a separate social layer, and four additional agent attributes are added to ensure the movement of agents. transCovasim follows the same rules of infection transmission dynamics as the base model. In this work, the Covasim model [16] was taken as the base agent-based model."
>
> **Methods, transCovasim**
>
> "All data and code used for running experiments and visualizing results, and the transCovasim source code are available online at https://github.com/KonstantinKlochkovv/transportation-motifs. transCovasim provides a framework for studying human mobility in agent-based

models, allowing agents to be exchanged between parallel simulations of the base agent-based model while preserving the initial social network structure within each population.“

Data and Code Availability Statement

Reviewer Comment

→ In the hub-and-spoke source identification, are symmetries holding back results. Because all satellite communities are equal in size and connectivity to Moscow, it would seem challenging to identify the source. Would the source be more identifiable if node sizes and/or connection weights were heterogeneous?

Response

We appreciate this suggestion. In accordance with the Reviewer's comment, we conducted additional experiments. We ran 150 simulations for each of the five cities in the hub-and-satellite system, using each city in turn as the outbreak origin and applying different traffic flow multipliers. We considered the symmetric system described in our article (660,000 agents in the hub and 110,000 in each of the satellites) and an asymmetric system (660,000 agents in the hub, 200,000 in the first satellite, and 80,000 in each of the others). The results are shown in the figure below. In the asymmetric case, the overall pattern of the dependence of accuracy on the day of the epidemic and the testing probability was preserved and the difference in the accuracy is relatively low.

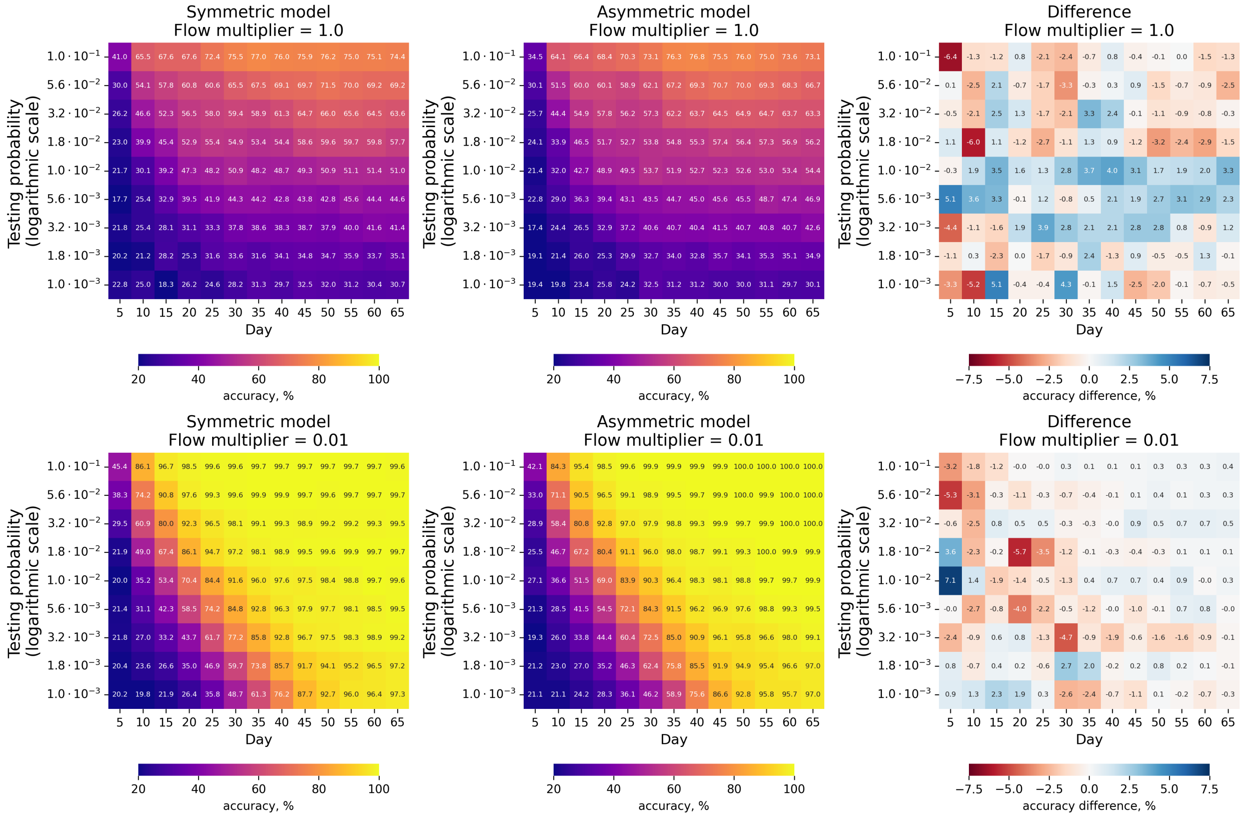

However, some patterns can be inferred. For systems with a traffic flow multiplier of 1, no clear effect is observed. When testing more than 1% of the population per day, the identification of the outbreak city is more accurate for the symmetric system, and when testing 1% or less, it is more accurate for the asymmetric system. In a system with such close transport links between cities, when an epidemic starts in a satellite, it quickly spreads to the hub, and the incidence in the hub becomes higher than in the satellite, which makes it harder to identify the city where the outbreak occurred. For this reason, the larger size of the first satellite in an asymmetric system allows for more accurate differentiation between the start of the epidemic in the satellite or in the hub when the number of people tested is small, which determines greater accuracy of identification. With a larger number of people tested, it becomes possible to identify any of the outbreak cities, and it becomes more important to identify all satellites uniformly, which is ensured by a symmetric system.

With a transport flow multiplier of 0.01, for any number of daily tests, the identification of the outbreak city is slightly more accurate for the symmetric system for the reason described above. Now there is no problem of rapid spread of the epidemic from the satellite to the hub, so the quality of detection is determined by the quality of outbreak detection in a particular city, which is ensured by the uniform distribution of agents across satellites, i.e., a symmetric system.

Thus, neither the asymmetric nor the symmetric system showed a clear overall advantage, as the outcome depended on the combination of conditions considered. Since these analyses did not reveal a consistent general pattern, they were not included in the main text of the manuscript.

Reviewer Summary

> Overall, I find this work interesting and potentially useful if oriented towards a "next pandemic" or disease X. The conclusions support interventions affecting population mobility. In practice, my understanding is that mobility restrictions have demonstrated mixed impact. The literature review focuses attention to the early outbreak in China and other simulation papers, but a few more examples here to illustrate where such interventions have worked/failed may help readers connect this work to practical policy.

Response

We appreciate your high evaluation of our work. In accordance with your comment, we have expanded the literature review in the Introduction section, adding examples of countries and regions where mobility restriction measures were and were not effective during the COVID-19 pandemic, as well as recommendations for optimizing such policies.

> "Human mobility interventions have proven effective when applied early in regions distant from the initial outbreak of the COVID-19 epidemic. Similar restrictions could have reduced the number of deaths in the islands macro-region of Italy by 12% if introduced almost a month earlier than they were, and by about 4% without early application of travel restrictions [17]. According to various estimates, international movement restrictions reduced the number of exported cases by 80% until mid-February 2020 [11,18,19]. On the other hand, mobility interventions did not show the same significant effect in mainland China, where the Wuhan travel ban delayed the epidemic progression by only

3–5 days [11, 20], and in Iran, where no significant effect on COVID-19 trends was observed as a result of a smart travel ban policy without other active measures [1]. For practical policy purposes, it is important to prioritize limiting long-distance connections [5, 6], implement restrictions early in the epidemic [12, 17], and combine mobility interventions with community-level non-pharmaceutical interventions [1, 11]. At the same time, it is necessary to weigh these epidemiological benefits against decline in health service use such as observed in the Democratic Republic of Congo during the COVID-19 pandemic [21].“

Introduction

References

1. Nassiri H, Mohammadpour SI, Dahaghin M. How do the smart travel ban policy and intercity travel pattern affect COVID-19 trends? Lessons learned from Iran. Plos one. 2022;17(10):e0276276.

2. Balcan D, Colizza V, Gonçalves B, Hu H, Ramasco JJ, Vespignani A. Multiscale mobility networks and the spatial spreading of infectious diseases. Proceedings of the national academy of sciences. 2009;106(51):21484-9.

3. Makhrova AG, Kirillov PL, Bochkarev AN. Work commuting of the population in the Moscow agglomeration: estimating commuting flows using mobile operator data. Regional Research of Russia. 2017;7(1):36-44.

4. Ye X, Zhu Y, Wang T, Yan X, Chen J, Zheng P. Assessing the Impact of Travel Restrictions on the Spread of the 2020 Coronavirus Epidemic: An Advanced Epidemic Model Based on Human Mobility. Sustainability. 2023;15(16):12597.

5. Xu XK, Liu XF, Wang L, Wu Y, Lu X, Wang X, et al. Assessing the spread risk of COVID-19 associated with multi-mode transportation networks in China. Fundamental Research. 2023;3(2):305-10.

6. Schlosser F, Maier BF, Jack O, Hinrichs D, Zachariae A, Brockmann D. COVID-19 lockdown induces disease-mitigating structural changes in mobility networks. Proceedings of the National Academy of Sciences. 2020;117(52):32883-90.

7. Pardo-Araujo M, García-García D, Alonso D, Bartumeus F. Epidemic thresholds and human mobility. Scientific reports. 2023;13(1):11409.

8. Le TM, Raynal L, Talbot O, Hambridge H, Drovandi C, Mira A, et al. Framework for assessing and easing global COVID-19 travel restrictions. Scientific Reports. 2022;12(1):6985.

9. Stenseth NC, Schlatte R, Liu X, Pielke Jr R, Li R, Chen B, et al. How to avoid a local epidemic becoming a global pandemic. Proceedings of the National Academy of Sciences. 2023;120(10):e2220080120.

10. Viboud C, Bjørnstad ON, Smith DL, Simonsen L, Miller MA, Grenfell BT. Synchrony, waves, and spatial hierarchies in the spread of influenza. science. 2006;312(5772):447-51.

11. Chinazzi M, Davis JT, Ajelli M, Gioannini C, Litvinova M, Merler S, et al. The effect of travel restrictions on the spread of the 2019 novel coronavirus (COVID-19) outbreak. Science. 2020;368(6489):395-400.

12. Ji H, Tong H, Wang J, Yan D, Liao Z, Kong Y. The effectiveness of travel restriction measures in alleviating the COVID-19 epidemic: evidence from Shenzhen, China. Environmental geochemistry and health. 2022;44(9):3115-32.

13. Brockmann D, Helbing D. The hidden geometry of complex, network-driven contagion phenomena. science. 2013;342(6164):1337-42.

14. Li J, Xiang T, He L. Modeling epidemic spread in transportation networks: A review. Journal of Traffic and Transportation Engineering (English Edition). 2021;8(2):139-52.

15. Barrett CL, Bisset KR, Eubank SG, Feng X, Marathe MV. Episimdemics: an efficient algorithm for simulating the spread of infectious disease over large realistic social networks. In: SC'08: Proceedings of the 2008 ACM/IEEE Conference on Supercomputing. IEEE; 2008. p. 1-12.

16. Kerr CC, Stuart RM, Mistry D, Abeysuriya RG, Rosenfeld K, Hart GR, et al. Covasim: an agent-based model of COVID-19 dynamics and interventions. PLOS Computational Biology. 2021;17(7):e1009149.

17. Parino F, Zino L, Porfiri M, Rizzo A. Modelling and predicting the effect of social distancing and travel restrictions on COVID-19 spreading. Journal of the Royal Society Interface. 2021;18(175).

18. Anzai A, Kobayashi T, Linton NM, Kinoshita R, Hayashi K, Suzuki A, et al. Assessing the impact of reduced travel on exportation dynamics of novel coronavirus infection (COVID-19). Journal of clinical medicine. 2020;9(2):601.

19. Wells CR, Sah P, Moghadas SM, Pandey A, Shoukat A, Wang Y, et al. Impact of international travel and border control measures on the global spread of the novel 2019 coronavirus outbreak. Proceedings of the National Academy of Sciences. 2020;117(13):7504-9.

20. Kraemer MU, Yang CH, Gutierrez B, Wu CH, Klein B, Pigott DM, et al. The effect of human mobility and control measures on the COVID-19 epidemic in China. Science. 2020;368(6490):493-7.

21. Hategeka C, Carter SE, Chenge FM, Katanga EN, Lurton G, Mayaka SMN, et al. Impact of the COVID-19 pandemic and response on the utilisation of health services in public facilities during the first wave in Kinshasa, the Democratic Republic of the Congo. BMJ Global health. 2021;6(7).
