# Supplementary material for: Human mobility and outbreak origins in epidemic spread: Insights from agent-based modeling
Source: PLoS Comput Biol. 2026 May 12;22(5):e1014279. doi: 10.1371/journal.pcbi.1014279 (PMC13186328; doi:10.1371/journal.pcbi.1014279)
Supplement: Attachment — Submitted filename: Response_to_Reviewers_auresp_2.pdf [file pcbi.1014279.s006.pdf]

# Response To Reviewer #1

---

## Reviewer Comment

> The authors have addressed most of my concerns in this revision.
>
> However, I am still worried about model heterogeneity.
>
> Line 301-310: Several limitations mentioned here are all related to model heterogeneity, but they are described less specific. Could you please elaborate on the heterogeneity issues brought by different situations that you mentioned here, and add future research directions or plans to better list appropriate solutions?

## Response

We appreciate this suggestion. In accordance with the Reviewer's comment, we have added a more detailed description of the limitations of our study related to the heterogeneity of traffic flows and their implications, as well as our plans to address these issues in future work.

> "However, our study has several limitations related to the heterogeneous nature of traffic flows. First, we do not account for the coexistence of multiple transport modes. In real-world systems, mobility occurs across road, bus, rail, and air networks, each characterized by distinct topologies, temporal and spatial scales, and user populations, which may themselves differ in socioeconomic and behavioral attributes [1–3]. These differences can lead to substantially different patterns of connectivity and transmission potential across modes.
>
> Second, travel is not instantaneous and may itself contribute to disease transmission through close contacts between passengers. The intensity and structure of such contacts depend strongly on the mode of transport, introducing additional heterogeneity that is not captured in our framework.
>
> Third, the purpose of travel (e.g., commuting vs. tourism) introduces systematic variation in movement patterns and contact structures. Long-distance tourist trips often involve small, cohesive groups (e.g., families), whereas commuting typically exhibits strong spatiotemporal regularity, with repeated trips along fixed routes and stable contact patterns that occur primarily at destinations such as workplaces rather than during transit [4–6]. Neglecting these forms of regularity may lead to an overestimation of effective population mixing during travel and, consequently, to inflated estimates of transmission rates.
>
> Finally, in systems with multiple transport modes, interventions targeting a single mode may induce adaptive redistribution of flows to alternative modes, thereby reducing the overall effectiveness of such measures. This substitution effect is not captured in the present model.
>
> In future work, we plan to address these limitations by incorporating mode-specific mobility layers, explicitly modeling transmission within transport-specific contact networks, and accounting for the regularity and purpose of individual movement patterns. Achieving this level of detail, particularly at the country scale, will require high-resolution mobility data, such as mobile phone records or detailed travel surveys [7].

Developing robust methods for integrating such data while maintaining model tractability remains an important direction for further research.“

**Conclusion and discussion**

## References


1. Xu XK, Liu XF, Wang L, Wu Y, Lu X, Wang X, et al. Assessing the spread risk of COVID-19 associated with multi-mode transportation networks in China. Fundamental Research. 2023;3(2):305-10.

2. Zheng R, Xu Y, Wang W, Ning G, Bi Y. Spatial transmission of COVID-19 via public and private transportation in China. Travel medicine and infectious disease. 2020;34:101626.

3. Balcan D, Colizza V, Gonçalves B, Hu H, Ramasco JJ, Vespignani A. Multiscale mobility networks and the spatial spreading of infectious diseases. Proceedings of the national academy of sciences. 2009;106(51):21484-9.

4. Giles JR, zu Erbach-Schoenberg E, Tatem AJ, Gardner L, Bjørnstad ON, Metcalf C, et al. The duration of travel impacts the spatial dynamics of infectious diseases. Proceedings of the National Academy of Sciences. 2020;117(36):22572-9.

5. Danon L, House T, Keeling MJ. The role of routine versus random movements on the spread of disease in Great Britain. Epidemics. 2009;1(4):250-8.

6. Gonzalez MC, Hidalgo CA, Barabasi AL. Understanding individual human mobility patterns. nature. 2008;453(7196):779-82.

7. Wesolowski A, Buckee CO, Engø-Monsen K, Metcalf CJE. Connecting mobility to infectious diseases: the promise and limits of mobile phone data. The Journal of infectious diseases. 2016;214(suppl_4):S414-20.
